# A Novel Association Between Coronavirus Disease 2019 and Normocomplementemic Rapidly Progressive Glomerulonephritis-Crescentic Immunoglobulin A Nephropathy: A Report of Two Pediatric Cases

**DOI:** 10.7759/cureus.22077

**Published:** 2022-02-10

**Authors:** Vivekanand N, R. K. Naresh Singh, Neha Kumari, Raksha Ranjan, Sandeep Saini

**Affiliations:** 1 Pediatrics, All India Institute of Medical Sciences, Rishikesh, Rishikesh, IND; 2 Pathology, All India Institute of Medical Sciences, Rishikesh, Rishikesh, IND; 3 Pediatrics and Neonatology, Adesh Institute of Medical Sciences and Research, Bathinda, IND; 4 Pediatrics and Pediatric Nephrology, All India Institute of Medical Sciences, Rishikesh, Rishikesh, IND; 5 Nephrology, All India Institute of Medical Sciences, Rishikesh, Rishikesh, IND

**Keywords:** immunoglobulin a nephropathy, covid-19, pediatric, glomerulonephritis, kidney biopsy

## Abstract

Coronavirus disease 2019 (COVID-19), caused by the severe acute respiratory syndrome coronavirus 2 (SARS-CoV-2), has been predominately associated with respiratory illness. Acute kidney injury (AKI) is the most common reported kidney involvement. Kidney complications, including proteinuria, hematuria, and rarely collapsing glomerulopathy (CG), a form of focal segmental glomerulosclerosis (FSGS), are also well known now and are frequently documented in the literature published so far.

We present two cases of glomerulonephritis (GN) in the setting of AKI in COVID-19 infection in children. Kidney biopsy specimens showed immunoglobulin A nephropathy (IgAN) with crescentic GN (CGN) with acute tubular injury with focal medium artery vasculitis. The patients exhibited a severe presentation and rapid progression to end-stage renal disease (ESRD). This report attempts to add a bit to the evolving information on COVID-19 disease, especially in children as far as kidney involvement is concerned.

## Introduction

Across countries, coronavirus disease 2019 (COVID-19) caused by the novel severe acute respiratory syndrome coronavirus 2 (SARS-CoV-2) is not a uniform phenomenon; some suffer severe illness, and some are spared. The pandemic still has time to last and manifest. In India, the COVID-19 disease is less severe among the pediatric population, with fewer children requiring hospitalization and intensive care unit (ICU) admissions.

The principal target organ system of COVID-19 is the respiratory system, with severe manifestations such as respiratory distress syndrome and diffuse alveolar hemorrhage. Angiotensin-converting enzyme 2 (ACE2) expressed in the lungs has been proposed as the target for SARS-CoV-2 for binding to the host cell surface and internalization. ACE2 is also expressed in the kidney (in proximal tubules and podocytes) and is a target for SARS-CoV-2 [1]. The other theories proposed are acute tubular necrosis, protein leakage, and collapsing glomerulopathy (CG) due to direct infection of kidney podocytes and proximal tubular cells. Following SARS-CoV-2 illness, several alterations in innate and adaptive immune responses are reported. Immunosenescence reported in COVID-19 resulting in inefficient viral clearance, enhanced release of cytokines and chemokines, endothelial damage, and activation of the coagulation and complement cascades is one such mechanism [2].

According to a review, acute kidney injury (AKI) could occur in <10% of COVID-19 cases, with 44% of patients presenting with proteinuria, 26.7% with hematuria, and 15.5% with elevated serum creatinine [3]. SARS-CoV, Middle Eastern respiratory syndrome (MERS) CoV, and SARS-CoV-2 seem to cause fewer symptoms and less severe disease in children compared with adults and are associated with much lower case fatality rates. However, children more often have gastrointestinal symptoms than adults [3]. The current knowledge about the disease is still evolving, with new literature being published each passing day.

We hypothesize that coronavirus is the second hit in immunoglobulin A nephropathy (IgAN) leading to immune complex-mediated disease and may have clinical presentation as severe as rapidly progressive glomerulonephritis (RPGN) and end-stage renal disease (ESRD). To the best of our knowledge, we report the first-ever cases of RPGN-IgAN so far due to SARS-CoV-2 in the pediatric population.

## Case presentation

Case 1

**Figure 1:**
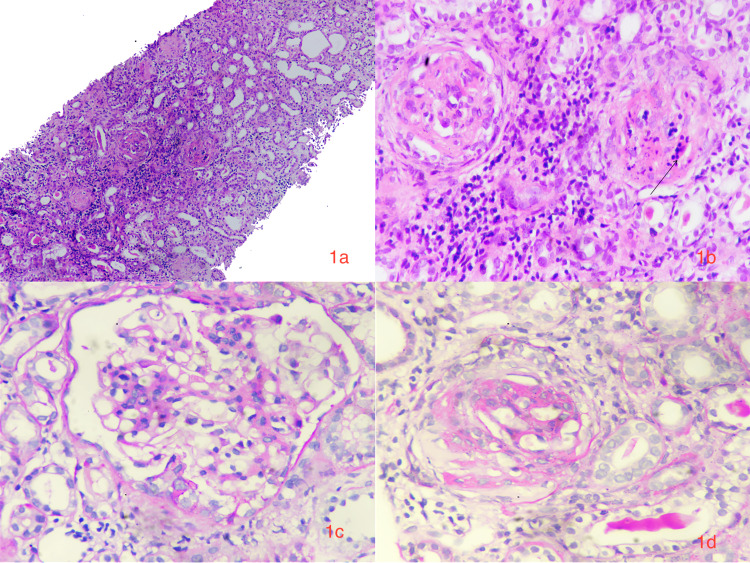
1a: Renal cortical tissue with glomeruli and inflamed tubulointerstitium (hematoxylin and eosin (H&E), 10×). 1b: Cellular crescent with necrotizing lesion characterized by karyorrhectic debris, disruption of capillary walls, and bright eosinophilic fibrin deposition in the underlying glomerular tuft (marked by arrow). Moderate mononuclear infiltrate in the interstitium (periodic acid–Schiff (PAS), 40×). 1c: Areas showing mesangial hypercellularity and matrix expansion (PAS, 40×). 1d: Fibrocellular crescents with disruption of Bowman’s capsule (PAS, 40×).

**Figure 2:**
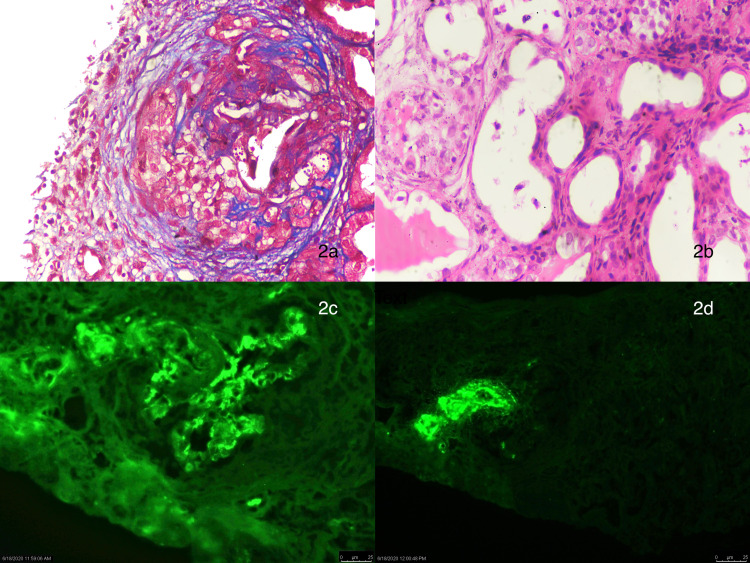
2a: Massive cellular crescents with peri-glomerular fibrosis (Masson trichrome, 40×). 2b: Acute tubular injury (cystic dilation of tubules with flattening and desquamation of epithelial cells, disruption of brush border, and tubular basement membrane) (H&E, 20×). 2c: Immunofluorescence showing strong staining of mesangium with IgA (3+/4+). 2d: Immunofluorescence showing deposition of fibrinogen in the vessel wall (3+). There is fibrinoid necrosis of the vessel wall, indicating vasculitis.

A 13-year-old male from a northern state of India presented with vomiting, headache, reduced urine output, and generalized anasarca in the past 10 days. The patient gave a negative history of fever, cough, sore throat, runny nose, abdominal pain, diarrhea, painful micturition, fatigue, rash, photosensitivity, recent drug exposure, chronic illness, and upper respiratory tract infection symptoms in other family members. The patient was afebrile, with a heart rate of 84 beats/minute, respiratory rate of 26 breaths/minute, oxygen saturation (S_P_O_2_) of 98% on room air, and blood pressure (BP) of 140/100 mm Hg (>95th centile). Physical examination was remarkable for mild pallor and bilateral (B/L) pitting pedal edema. Examination of the heart, lungs, and abdomen was unremarkable. His weight (Wt) was 62 kg (pre-illness Wt: 52 kg) and height (Ht) was 161.5 cm (median to +1 standard deviation (SD)). Blood workup and urine analysis (UA) are outlined in Table 1. An X-ray of the chest showed an unremarkable B/L lung field. A kidney ultrasound revealed normal size kidneys with no loss of corticomedullary differentiation. Serum lactate dehydrogenase (LDH) was elevated with low haptoglobin, and a peripheral blood smear was normocytic to normochromic with no evidence of fragmented red blood cells (RBCs) or schistocytes. Direct Coombs test (DCT)/indirect Coombs test (ICT) and D-dimer were negative. C-reactive protein (CRP) and procalcitonin were normal. Complement levels were normal with negative antineutrophil cytoplasmic antibody (ANCA) and antinuclear antibody (ANA). The COVID-19 reverse-transcriptase polymerase chain reaction (RT-PCR) assay came positive for the patient. The preliminary reports were suggestive of AKI with nephritic features, and methylprednisolone pulse followed by oral prednisolone was given. Hemodialysis was required within days of admission for oliguria and fluid overload. A kidney biopsy was performed for severe AKI with nephritis with hypertension. It was suggestive of IgAN with crescentic glomerulonephritis (Figure 1 and Figure 2), with global glomerulosclerosis (8/30), and segmental glomerulosclerosis (2/30) with minimal interstitial and tubular atrophy (5% cortex) with focal vasculitis in two medium arteries (M1 E1 S1 T0 C2) with no evidence of fibrosis. The child received three cycles of cyclophosphamide and regular hemodialysis.

**Table 1:** Analyte trends throughout hospitalization in case 1. TLC: total leukocyte count; GFR: glomerular filtration rate; RBCs: red blood cells; HPF: high-power field

Analyte	Results								Reference range
	D1	D5	D10	D15	D20	D25	D35	D45	
Hemoglobin (g/dL)	7.1	7.3	8	5.29	6.4	5.6	6.2	7.5	11.5–15.5
TLC (cells/mm^3^)	7,353	9,760	14,600	21,080	19,450	14,430	16,190	13,400	4,500–13,500
Platelet count (10^3^/µL)	56.5	120	123	61.88	85	182	201	118	150–350
Serum creatinine (mg/dL)	20.6	8.8	7.6	5.1	5.4	5.2	5.3	4.7	0.5–1.0
Blood urea (mg/dL)	199.8	130.3	146.5	96.3	135.3	115	85	100	5–18
GFR (mL/minute/1.73 m^2^)	3.3	-	-	-	-	-	-	-	>60
Serum albumin (g/dL)	4.36	3.84		4.1	4.2	4.1	4.0	4.1	3.6–5.2
Serum sodium (mEq/L)	138	126.6	128.3	130.2	130.1	134.6	129	130	135–147
Serum potassium (mEq/L)	6.3	5.1	5.4	4	3.3	3.16	3	3.7	3.5–5.1
Serum calcium (mg/dL)	8.73	8.2	8.26	9.1	8.7	8.4	8.6	8.6	8.4–10.2
Serum chloride (mEq/L)	99	92	91.6	93.4	95.4	95	97	99	97–107
Urine protein	3+	2+	Nil	Nil	-	-	-	-	Nil
Urine RBCs (cells/HPF)	5–7	5–6	1–3	1–2	-	-	-	-	Nil

Based on a presentation similar to our case, other possibilities considered and kept in mind while evaluating and managing and were not forgotten are hemolytic uremic syndrome (HUS), Henoch-Schonlein purpura (HSP), thrombotic thrombocytopenic purpura (TTP), post-streptococcal GN, rhabdomyolysis, vasculitis, and systemic lupus erythematosus (SLE).

Case 2

A 16-year-old male from a northern state of India presented with reduced urine output and generalized body swelling off and on for the past three months. There was a history of diarrhea one week before the onset of swelling. The patient was afebrile, with a heart rate of 88 beats/minute, respiratory rate of 26 breaths/minute, S_P_O_2_ of 98% on room air, and BP of >95th centile. Physical examination was remarkable for B/L pitting pedal edema. There was reduced air entry in B/L basal lung fields, with mild abdominal distention due to fluid. His Wt was 46 kg (pre-illness Wt: not known) and Ht was 156 cm (-1 to -2 SD). UA and blood workup are outlined in Table 2. An X-ray of the chest showed B/L pleural effusion. Kidney ultrasound showed bilateral moderately raised renal cortical echogenicity. Peripheral smear was normocytic to normochromic with no evidence of fragmented RBCs or schistocytes; serum LDH was normal. DCT/ICT and D-dimer were negative. CRP and procalcitonin were normal. Complement levels were normal with negative ANA. The COVID-19 RT-PCR assay was negative for the patient. Anti-COVID-19 IgG antibody was reactive; IgM was nonreactive. Serum creatinine level was progressively increasing with significant fluid overload and AKI. A kidney biopsy was performed, which showed features suggestive of IgA nephropathy with crescentic glomerulonephritis, global glomerulosclerosis and segmental glomerulosclerosis, focal acute tubular injury with degenerative changes, minimal interstitial fibrosis, and tubular atrophy. The patient received methylprednisolone pulse therapy, followed by oral prednisolone and hemodialysis. As there was no response to methylprednisolone, the patient has received cyclophosphamide and is on regular hemodialysis.

**Table 2:** Analyte trends throughout hospitalization in case 2. TLC: total leukocyte count; GFR: glomerular filtration rate; RBCs: red blood cells; HPF: high-power field; PCR: protein/creatinine ratio

Analyte	Results							Reference range
	D1	D5	D10	D15	D20	D25	D35	
Hemoglobin (HB) (g/dL)	7.8	8.5	8.6	7.7	9.8	8.5	8	11.5–15.5
TLC (cells/mm^3^)	8,074	8,340	13,900	22,950	14,180	10,370	9,070	4,500–13,500
Platelet count (10^3^/µL)	142	315	379	210	150	146	206	150–350
Serum creatinine (mg/dL)	5.89	7.14	6.48	4.2	3.2	3.5	3.3	0.5–1.0
Blood urea (mg/dL)	90	128	156	156	118	177	176	5–18
GFR (mL/minute/1.73 m^2^)	10.9	-	-	-	-	-	-	>60
Serum albumin (g/dL)	2.46	-	-	-	3.19	-	-	3.6–5.2
Serum sodium (mEq/L)	141.8	130	130	131	137	138	140	135–147
Serum potassium (mEq/L)	5.4	6.5	5.3	4.8	4.7	4.9	4.8	3.5–5.1
Serum calcium (mg/dL)	7.09	7.78	7.7	7.8	8	8.4	7.9	8.4–10.2
Serum chloride (mEq/L)	100	98	93	93	96	96	97	97–107
Urine protein	2+	2+	-	-	-	3+	-	Nil
Urine RBCs (cells/HPF)	10–12	50	-	-	-	20–25	-	Nil
Urine PCR	8.5	-	-	-	-	-	-	-

## Discussion

The kidney is one of the target organs of immune system dysregulation, whether primary or due to systemic disease, and the clinicopathological manifestation is GN. Recent advances in our understanding of the pathophysiological mechanisms suggest the assessment of biomarkers genetic analysis along with immunological features. Unfortunately, data on pediatric GN is scarce and often derived from adult studies. Similarly, significantly less data is available regarding COVID-19-associated nephropathy (COVAN) in children.

In adult patients with COVID-19, the most common COVID-19-associated nephropathy (COVAN) reported is CG, which seems to occur mainly in patients with non-severe respiratory symptoms of COVID-19 and isolated AKI or those presenting with glomerular proteinuria [4]. Other histological findings are acute tubular injury, endothelial injury, or thrombotic microangiopathy [5].

The GN diagnosis in both cases is based on the clinical presentation of rapid and progressive AKI with proteinuria, microscopic hematuria, and significant hypertension in the setting of RT-PCR-confirmed COVID-19 disease in case 1 and positive IgG COVID-19 antibodies in case 2. Kidney biopsy with immunofluorescence suggests IgA-mediated immune deposits in the form of crescents known as crescentic-IgAN (C-IgAN). According to the Oxford classification [6], renal biopsy showed cellular or fibrocellular crescents (C2) with hypercellular mesangium (M1) and endocapillary hypercellularity (E1), along with segmental and global glomerulosclerosis, with features suggestive of vasculitis in two medium arteries in case 1 and tubular atrophy in case 2.

When manifesting as a progressive rapid decline in renal function over a short period, GN is RPGN. Immune complex-RPGN is the most severe clinical and histological form, particularly in children with postinfectious GN, IgAN, IgA vasculitis, and lupus nephritis. The pathognomonic histological picture is crescents in more than 30% of the glomeruli, usually found in adults and uncommonly associated with IgAN.

IgAN or Berger’s disease, first described in 1968 by Berger and Hinglais, is the most common primary GN throughout the world in adolescents and young adults, with a male predominance. Recently, IgAN was redefined as an immune complex-mediated disease with multihit pathogenesis [7]. IgAN usually presents as synpharyngitic hematuria or recurrent gross hematuria or incidentally detected as microscopic hematuria, with pain abdomen with or without mild proteinuria. However, in less than 10% of cases, progression is rapid or fulminant, with generalized edema, AKI with or without oliguria, and hypertension [8].

From a retrospective, observational study by Shima et al. on 515 children diagnosed with IgAN, the prevalence of C-IgAN was 4.9% (25 cases). Among the 25 children with C-IgAN, 16 patients were asymptomatic. Incidentally, they detected from screening program, and the 13-year survival of the C-IgAN cases was worse than non-C-IgAN, with a low renal survival curve (77% versus 92.6%, p < 0.001) [9].

Literature regarding kidney dysfunction and injury secondary to SARS-CoV-2 is still evolving, and there is a shortage of literature in the pediatric population; hence, the true incidence remains unknown. Tubular damage due to cytokine storm, a direct cytopathic effect, and immune-mediated glomerulonephritis are possible causes considered for kidney involvement by COVID-19; the exact mechanism has not been fully elucidated yet [10].

The incidence of AKI associated with COVID-19 across the globe has been inconsistent, with initial Chinese studies showing an incidence of 0.5%, and the requirement of CRRT among them was 0.8%, which was drastically different from the recent report from the large cohort of 5,449 hospitalized COVID-19 cases, of which 1,993 (31.1%) cases had stage 1 AKI, with 14.3% requiring CRRT [11]. This discrepancy in the incidence of AKI is explained by the heterogeneity in the population, the definition of AKI followed, a requirement of ICU, underlying dormant renal pathology, associated comorbidities in the cases recruited, and COVID-19 being a nonuniform phenomenon across the continents.

Autopsy reports have demonstrated the existence of tropism of the virus to the glomerular epithelial cells and podocytes via ACE2 receptor, by which the virus gains entry and multiply [1]. There have been reports of glomerulosclerosis and high-risk factors such as apolipoprotein 1 (APOL-1) allele in COVID-19-positive cases [12]. The detection of viral antigens, inclusion bodies, and viral particles in the epithelial cells and podocytes of the renal glomeruli and isolation of SARS-CoV-2 in urine samples can positively explain the possibility of a COVAN. One similar case was reported from Iran of COVID-19-associated CGN in an adult patient who had responded well to medical therapy and did not require continuous renal replacement therapy [13].

Our cases had progressive deterioration of renal function and received pulse methylprednisolone, followed by oral prednisolone and hemodialysis. No evidence-based treatment guideline for pediatric IgAN is available. In 2006, Appel concluded that there is no consensus on the best treatment [14]. The combination of renin-angiotensin-aldosterone system (RAAS) blockers and corticosteroids should be the treatment for patients with normal renal function and moderate proteinuria [15] and cyclophosphamide for rapidly progressive IgAN [16]. Rituximab is not effective in patients with IgAN. Eculizumab has shown promising results in a few patients [6]. Tonsillectomy is also suggested, but not widely accepted as a treatment [17].

## Conclusions

The rapid progression of renal insufficiency in both cases could be attributed to the pathological findings in kidney biopsy with C2, M1, and E1, along with glomerulosclerosis and medium vessel vasculitis. Furthermore, the absence of the features of chronic GN, such as tubular atrophy or interstitial fibrosis (T0) and medium vessel vasculitis strongly, associates it to a recent insult, the SARS-CoV-2 virus. SARS-CoV-2, which is known to cause hemodynamic changes secondary to cytokine storm and ensuing capillary leak syndrome, might precipitate or aggravate RPGN. C-IgAN due to SARS-CoV-2 or any other etiology can be refractory to treatment moving toward ESRD. In such cases, various treatment dilemmas are still existing. The effectiveness of immunosuppression and plasmapheresis, and the role of renal transplant are unclear. Pediatricians should be aware of such atypical presentations and association with COVID-9 while tending to patients in this current pandemic.
